# Steroid Sulfation in Neurodegenerative Diseases

**DOI:** 10.3389/fmolb.2022.839887

**Published:** 2022-02-23

**Authors:** Jana Vitku, Martin Hill, Lucie Kolatorova, Eva Kubala Havrdova, Radmila Kancheva

**Affiliations:** ^1^ Department of Steroids and Proteofactors, Institute of Endocrinology, Prague, Czechia; ^2^ Department of Neurology and Center of Clinical Neuroscience, First Faculty of Medicine, Charles University and General University Hospital in Prague, Prague, Czechia

**Keywords:** steroid sulfotransferases, steroid sulfatase, neuroactive steroids, neurosteroids, brain, Alzheimer’s disease, Parkinson's disease, multiple sclerosis

## Abstract

Steroid sulfation and desulfation participates in the regulation of steroid bioactivity, metabolism and transport. The authors focused on sulfation and desulfation balance in three neurodegenerative diseases: Alzheimer´s disease (AD), Parkinson´s disease (PD), and multiple sclerosis (MS). Circulating steroid conjugates dominate their unconjugated counterparts, but unconjugated steroids outweigh their conjugated counterparts in the brain. Apart from the neurosteroid synthesis in the central nervous system (CNS), most brain steroids cross the blood-brain barrier (BBB) from the periphery and then may be further metabolized. Therefore, steroid levels in the periphery partly reflect the situation in the brain. The CNS steroids subsequently influence the neuronal excitability and have neuroprotective, neuroexcitatory, antidepressant and memory enhancing effects. They also exert anti-inflammatory and immunoprotective actions. Like the unconjugated steroids, the sulfated ones modulate various ligand-gated ion channels. Conjugation by sulfotransferases increases steroid water solubility and facilitates steroid transport. Steroid sulfates, having greater half-lives than their unconjugated counterparts, also serve as a steroid stock pool. Sulfotransferases are ubiquitous enzymes providing massive steroid sulfation in adrenal *zona reticularis* and *zona fasciculata.*. Steroid sulfatase hydrolyzing the steroid conjugates is exceedingly expressed in placenta but is ubiquitous in low amounts including brain capillaries of BBB which can rapidly hydrolyze the steroid sulfates coming across the BBB from the periphery. Lower dehydroepiandrosterone sulfate (DHEAS) plasma levels and reduced sulfotransferase activity are considered as risk factors in AD patients. The shifted balance towards unconjugated steroids can participate in the pathophysiology of PD and anti-inflammatory effects of DHEAS may counteract the MS.

## Introduction

Steroids are important components of endogenous signaling in the organism. Steroidogenesis takes place prominently in peripheral glands such as adrenals, gonads, placenta. However, some other tissues and organs, including the brain are also able to metabolize cholesterol to steroid molecules and exert effects in autocrine and paracrine manner (Labrie, 1991; Pluchino et al., 2019; Giatti et al., 2020a). The group of steroids that can influence actions in nervous system are called neuroactive steroids (NAS), with the subgroup of steroids–neurosteroids—that act in the nervous system and are also synthesized there (Corpéchot et al., 1981).

Under physiological conditions, NAS influence a broad spectrum of functions, such as brain development, sexual behavior, stress response, emotions, memory, and cognition (Vallée et al., 1997; Serra et al., 2000; Frye, 2001; Darnaudéry et al., 2002; Johansson et al., 2002; Hampl et al., 2015). In various pathophysiological states of the central nervous system (CNS), such as epilepsy, depression, anxiety and neurodegenerative diseases neuroactive steroid levels are altered (Hillen et al., 2000; Kancheva et al., 2010; Hill et al., 2011; MacKenzie and Maguire, 2013; Kanceva et al., 2015).

The most discussed NAS in humans include metabolites of progesterone, i.e., pregnanolone isomers, 5α/β-reduced metabolites of cortisol, sulfates of pregnanolone isomers, dehydroepiandrosterone sulfate (DHEAS) and pregnenolone sulfate (PregS). Furthermore, classical steroid hormones such as 17β-estradiol (E2) (Xu et al., 2008; Jiang et al., 2009) testosterone (Park-Chung et al., 1999), and progesterone (Wu et al., 1990) can act as NAS in the brain (reviewed in (Giatti et al., 2019)).

Biosynthesis of NAS, especially pregnane steroids, shows marked differences depending on the gender, age, menstrual cycle and pregnancy status (Kancheva et al., 2007). In premenopausal women, the largest proportion of pregnanolone isomers arise from progesterone synthesized in corpus luteum with the dominant metabolite allopregnanolone (3α-hydroxy-5α-pregnan-20-one) (Hill et al., 2005; Ottander et al., 2005; Hill and Očenášková, 2007). Gonadal pregnane may easily surpass the blood-brain barrier (BBB) as the brain concentrations reflect the ovarian production (Bixo et al., 1997).

Outside of pregnancy and the luteal phase of the menstrual cycle, the adrenal cortex produces most NAS and their precursors. Human z*ona fasciculata* produces PregS (Hill and Očenášková, 2007) in relatively high concentrations. In addition, *zona fasciculata* synthesizes mainly cortisol whose 5α/β-reduced metabolites can act as NAS (Strömberg et al., 2005); *zona reticularis* primarily produces massive amounts of DHEAS (Mueller et al., 2015).

Additionally, the CNS can synthesize neurosteroids directly in the CNS or from peripheral precursors (Corpéchot et al., 1981; Corpéchot et al., 1983; Luchetti et al., 2011). Maintaining a pool of the bioavailable steroids in the site of action is a dynamic process where different steroidogenic enzymes are involved. One of the systems regulating the activity of steroids are sulfotransferases (SULTs) and steroid sulfatase (STS), which add or detach sulfate moiety, respectively. This balance could be of importance in neurodegenerative processes as well as for the transport of soluble steroid conjugates to the respective active sites.

The role of unconjugated NAS in various neurodegenerative diseases were intensively reviewed recently (Melcangi et al., 2016; Yilmaz et al., 2019; Giatti et al., 2020b; Kudova, 2021) as well as in the past (Luchetti et al., 2011; Melcangi et al., 2008; Wang et al., 2001; Melcangi and Panzica, 2009; Melcangi et al., 2011; di Michele et al., 2003). In this review, we focused mainly on sulfated steroids and sulfation and desulfation pathways in three neurodegenerative diseases: Alzheimer´s disease (AD), Parkinson’s disease (PD), multiple sclerosis (MS), while the dysregulation of sulfation processes can change the bioavailability and activity of NAS and may influence the pathogenesis and progression of some diseases.

## Unconjugated Steroids

Unconjugated (free) steroids are predominantly lipophilic compounds, which can enter the cells and pass the BBB by simple nonsaturable diffusion. A major fraction of numerous steroids in circulation is bound to albumin. However, steroids can easily dissociate from the albumin complex and pass the BBB as well (Pardridge and Mietus, 1979; Pardridge and Mietus, 1980). Steroids that are bound to selective transport proteins (CBD-transcortin, SHBG-sex hormone binding globulin), are not transported through the BBB (Compagnone and Mellon, 2000). Part of steroids can be also synthesized *de novo* in CNS (Corpéchot et al., 1983). However, a substantial part of steroids can be synthesized from steroid precursors from periphery or can be transported from the periphery thus partly reflecting the circulating levels (Bixo et al., 1995; Kancheva et al., 2010; Kancheva et al., 2011). Therefore, the contribution of peripheral steroids to the steroid pool in CNS is important and changes in the steroid milieu in the CNS can subsequently have an impact on neuronal activity in brain (Vaňková et al., 2016). The steroid levels in cerebrospinal fluid (CSF) are generally lower than in circulation, while some CNS steroids (especially DHEA and some of its metabolites; allopregnanolone and cortisol) correlate with their peripheral levels (Kancheva et al., 2010).

**FIGURE 1:**
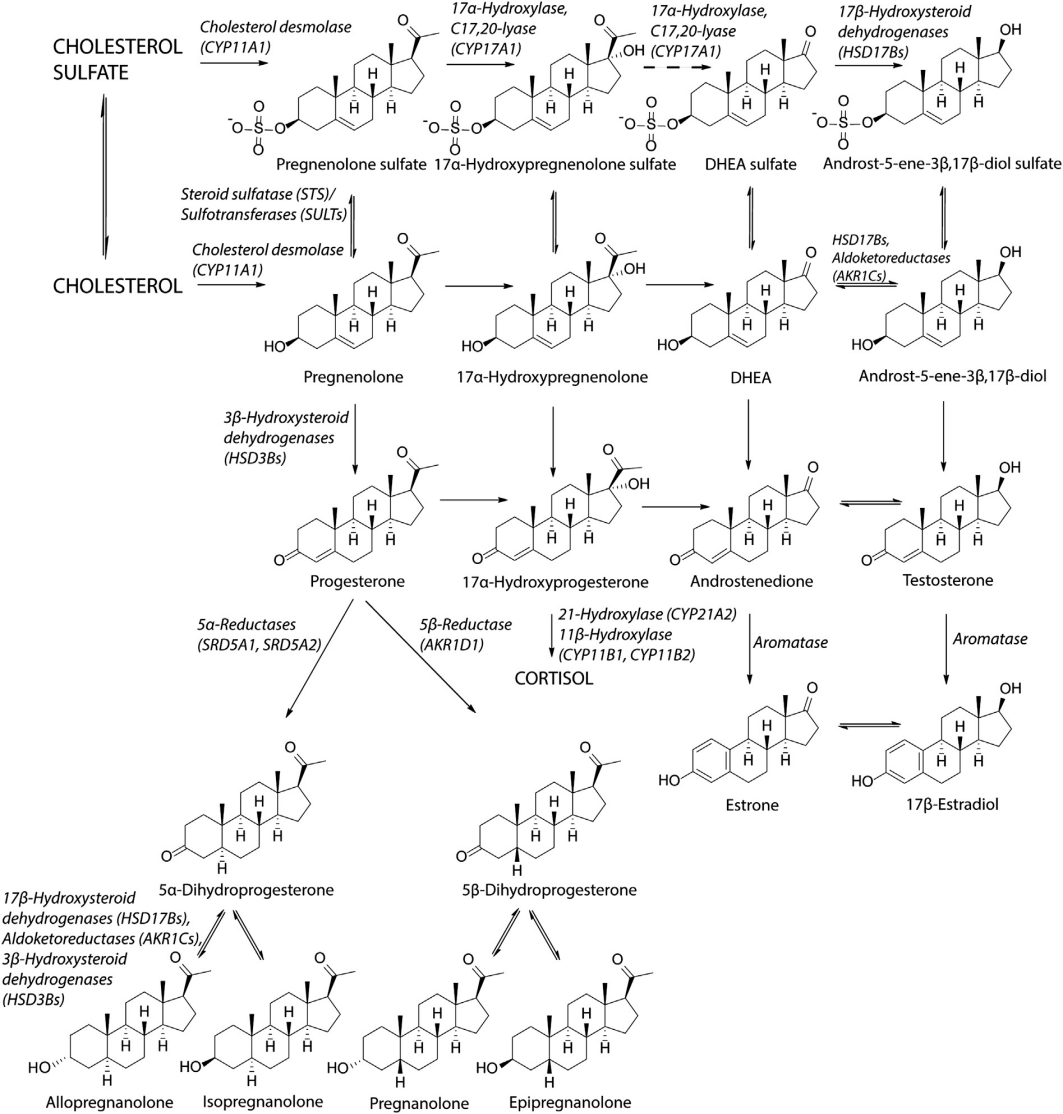
Scheme of steroidogenesis. Steroidogenic pathway for sulfated steroids is similar as the biosynthesis of unconjugated steroids. Cholesterol can be sulfated by sulfotransferase (SULT2B1b) to cholesterol sulfate (Fuda et al., 2002). Cholesterol sulfate can be converted to pregnenolone sulfate by cholesterol desmolase while cholesterol sulfate serves as a better substrate than unconjugated cholesterol (Tuckey, 1990). Pregnenolone sulfate can be subsequently converted to 17α-hydroxypregnenolone sulfate by CYP17A1 in the same way as pregnenolone (Neunzig et al., 2014). The lyase reaction of CYP17A1 has not been confirmed to take place by recent studies (Neunzig et al., 2014; Sánchez-Guijo et al., 2016; Neunzig and Bernhardt, 2018), although earlier studies indicated this conversion (Lamont et al., 1970; Jaffe et al., 1972). DHEAS can be converted to androst-5-ene-3β,17β-diol sulfate by 17β-hydroxysteroid dehydrogenases (Sánchez-Guijo et al., 2016; Qaiser et al., 2017). Regarding the unconjugated steroids, the reactions between DHEA and androst-5-ene-3β,17β-diol, androstenedione and testosterone and estrone and estradiol are preferentially conducted in the reductase direction (Purohit and Foster, 2012; Mostaghel, 2013).

Main unconjugated NAS include metabolites of progesterone (allopregnanolone, isopregnanolone, pregnanolone, epipregnanolone and pregnanediols), DHEA, E2, testosterone and their metabolites (androsterone, epiandrosterone, etiocholanolone, 3α-hydroxy-5α/β,17β-diols), 5α/β-reduced metabolites of glucocorticoids (3α-hydroxy, 5α/β-tetrahydro-cortisols, 3α-hydroxy-5α/β-tetrahydro-cortisones). Basic scheme of steroidogenesis is shown in Figure 1.

DHEA is mainly of adrenal origin, but it can also be synthesized in gonads (10–20%) (Nieschlag et al., 1973) and most probably also in the CNS (Stárka et al., 2015). DHEA is a substrate for testosterone production and subsequently for estrogen synthesis. These processes occur also in the human brain (Steckelbroeck et al., 2002) and may subsequently influence the nervous system by genomic as well as by non-genomic pathways. Furthermore, DHEA itself serves as neuroactive steroid (Stárka et al., 2015). DHEA has neuroprotective, anti-glucocorticoid, anti-apoptotic, anti-inflammatory and anti-oxidative properties, increases neurite growth and has impact on neurogenesis and catecholamine synthesis and secretion. However, it can also be neurotoxic (reviewed in (Maninger et al., 2009; Stárka et al., 2015)).

Pregnanolone isomers are progesterone metabolites originated through the action of ubiquitous 5α-reductase (SRD5As) and liver 5β-reductase (AKR1D1) forming 5α- and 5β-dihydroprogesterone, respectively (Havlíková et al., 2006). The subsequent metabolism to individual pregnanolone isomers is provided by a system of subfamily 1C aldoketoreductases (AKR1Cs) and 17β-hydroxysteroid dehydrogenases (HSD17Bs) (Miller and Auchus, 2011; Rižner and Penning, 2014; He et al., 2019) previously known as 3α- and 3β-hydroxysteroid oxidoreductases. Reduced metabolites of progesterone exert anxiolytic, sedative, hypnotic and anticonvulsive effects (Frye, 1995; Klein and Herzog, 1998; Hill et al., 2010).

Sex hormones are also active in the CNS. Particularly E2 exert pleiotropic effects there, facilitating learning and memory (Frick, 2015; Sun et al., 2019; Luine and Frankfurt, 2012; Tozzi et al., 2020; Dieni et al., 2020), as well as influencing emotional (Altemus, 2019) and sexual behavior (Diotel et al., 2018). They generally act as neuroprotective substances (Fargo et al., 2009; Spence and Voskuhl, 2012; Duong et al., 2020; Yang et al., 2020) and promote neurogenesis and neuro-regeneration (Pillerová et al., 2021; Azcoitia et al., 2019). These processes in the brain take place through classical nuclear steroid receptors (estrogen receptor α and β-ERα and ERβ, androgen receptor-AR, progesterone receptor-PR), non-classical membrane-associated steroid receptors (AR, ERα, ERβ) and transmembrane receptors (zinc transporter protein 9, G protein coupled estrogen receptor 1) (Pillerová et al., 2021). Finally, E2 can exert its action through neurotransmitter receptors such as serotonin receptor (Wetzel et al., 1998), L-type voltage gated channel (Sribnick et al., 2009; Vega-Vela et al., 2017; Sánchez et al., 2014) or N-methyl-D-aspartate (NMDA) receptor (Weaver et al., 1997; Foy et al., 1999). The action of E2 on L-type voltage channel as well as NMDA receptor appears to be concentration dependent (Table 1). Progesterone has also therapeutic benefits such as reduction of inflammation and edema, preventing myelin degradation and reducing excitotoxic neuronal death (Luoma et al., 2012).

**TABLE 1:** Positive and negative modulators of selected membrane receptors.

**Modulation of GABA**_**A**_ **receptor**			
Positive		Negative	
3α-OH-pregnanolone isomers (allopregnanolone, pregnanolone)	Majewska et al. (1986); Park-Chung et al. (1999); Strömberg et al. (2005)	3β-OH-pregnanolone isomers (isopregnanolone, epipregnanolone)	Wang et al. (2000), Wang et al. (2002); Lundgren et al. (2003)
3α-androstane steroids (androsterone, etiocholanolone, 5α-androstane-3α, 17β, diol)	Turner et al. (1989); Park-Chung et al. (1999); Kaminski et al. (2005)	PregS	Park-Chung et al. (1999); Majewska and Schwartz, (1987)
3α,5α/5β- THDOC	Majewska et al. (1986); Turner et al. (1989); Strömberg et al. (2005)	DHEAS	Park-Chung et al. (1999); Majewska et al. (1990)
weak: progesterone	Wu et al. (1990); Park-Chung et al. (1999)	Sulfates of pregnanolone isomers (pregnanoloneS, epipregnanoloneS, isopregnanoloneS)	Park-Chung et al. (1999)
weak: androstenedione	Park-Chung et al. (1999)	3β5β-THDOC	Wang et al. (2002)
weak: testosterone	Park-Chung et al. (1999)	sulfates of 5α-androstane isomers (androsteroneS and epiandrosteroneS)	Park-Chung et al. (1999)
weak: 11-keto-PregS	Park-Chung et al. (1999)	11β-hydroxy-PregS	Park-Chung et al. (1999)
—	—	weak: E2 sulfate	Park-Chung et al. (1999)
—	—	weak: DHEA	Park-Chung et al. (1999)
→ neuroinhibition		→ neuroactivation	
**Modulation of glycine receptor**			
Positive		Negative	
Allopregnanolone (nM-1 μM concentrations)	Weir et al. (2004); Jiang et al. (2006)	progesterone	Wu et al. (1990)
—	—	deoxycorticosterone	Wu et al. (1990)
—	—	17α-OH-progesterone	Wu et al. (1990)
—	—	corticosterone	Wu et al. (1997); Maksay et al. (2001)
—	—	pregS	Wu et al. (1997); Maksay et al. (2001)
—	—	DHEAS	Maksay et al. (2001)
—	—	pregnanolone	Jiang et al. (2006)
—	—	E2	Jiang et al. (2009)
—	—	testosterone	Bukanova et al. (2020)
—	—	epitestosterone	Bukanova et al. (2020)
—	—	5αDHT	Bukanova et al. (2020)
—	—	epiandrosterone	Bukanova et al. (2020)
—	—	dihydroandrostendione	Bukanova et al. (2020)
—	—	androstenedione	Bukanova et al. (2020)
—	—	androstendiol	Bukanova et al. (2020)
—	—	DHEA	Bukanova et al. (2020)
—	—	etiocholanedione	Bukanova et al. (2020)
—	—	Allopregnanolone (40 μM concentration)	Fodor et al. (2006)
→ neuroinhibition		→ neuroactivation	
**Modulation of sigma (σ) 1 receptor**			
Positive		Negative	
DHEAS	Monnet et al. (1995)	PregS	Monnet et al. (1995)
—	—	progesterone	Monnet et al. (1995)
→ neuroactivation		→ neuroinhibition	


Modulation of NMDA receptor			
Positive		Negative	
Sulfates of 5α-pregnanolone isomers	Weaver et al. (2000)	Sulfates of 5β-pregnanolone steroids (pregnanoloneS)	Yaghoubi et al. (1998); Park-Chung et al. (1994); Weaver et al. (2000)
PregS	Wu et al. (1991);Irwin et al. (1992)	E2 (μM concentrations)	Weaver et al. (1997)
DHEAS	Park-Chung et al. (1994)	—	—
E2 (pM-nM concentrations)	Foy et al. (1999)	—	—
→ neuroactivation		→ neuroinhibition	
**Modulation of kainate receptor**			
Positive		Negative	
progesterone	Wu and Chen, (1997)	PregS	Wu et al. (1991)
—	—	PregnanolonS	Park-Chung et al. (1994)
→ neuroactivation		→ neuroinhibition	
**Modulation of AMPA receptor**			
Positive		Negative	
—	—	pregS	Wu et al. (1991);Shirakawa et al. (2005)
—	—	pregnanolonS	Park-Chung et al. (1994)
—	—	→ neuroinhibition	
**Modulation of voltage-gated ion channels (L-type)**			
Positive		Negative	
E2 (pM concentrations)	Wu et al. (2005);Sarkar et al. (2008)	E2 (nM concentration)	Brewer et al. (2009); Sribnick et al. (2009); Sánchez et al. (2014)
—	—	allopregnanolone	Hu et al. (2007); Earl and Tietz, (2011)
—	—	progesterone	Luoma et al. (2012)
→ neuroactivation		→ neuroinhibition	
**Modulation of serotonin (5-HT3) receptor**			
Positive		Negative	
—	—	E2	Wetzel et al. (1998)
—	—	progesterone	Wetzel et al. (1998); Wu et al. (2000)
—	—	testosterone	Wetzel et al. (1998)
—	—	allopregnanolone	Wetzel et al. (1998)
—	—	→ neuroinhibition	
**Modulation of transient receptor potential (TRP) ion channels**			
Positive		Negative	
PregS (TRPM3)	Wagner et al. (2008)	E2 (TRPV1)	Xu et al. (2008)
—	—	DHEA (TRPV1)	Chen et al. (2004)
—	—	progesterone (TRPM3)	Majeed et al. (2012)
→ neuroactivation		→ neuroinhibition	

Abbreviations: DHEA, dehydroepiandrosterone; DHEAS, dehydroepiandrosterone sulfate; E2, 17β-estradiol; PregS, pregnenolone sulfate; THDOC, tetrahydrodeoxycorticosterone.

## Conjugated Steroids

Conjugated steroids predominantly include steroid sulfates and glucuronides. Namely the sulfates have an important role in the regulation of steroid metabolism and transport. Steroid sulfates are hydrophilic compounds. Therefore, their passive diffusion through BBB is limited when compared with their unconjugated counterparts. Organic anion transporters (OATs) belonging to the solute carrier (SLC) transporters superfamily are the primary transporters for cellular influx of steroid sulfates. On the other hand, multidrug resistance proteins (MRP) from the ATP-binding cassette (ABC) transporter superfamily provides efflux of steroid sulfates (Sodani et al., 2012; Mueller et al., 2015). Steroid sulfates are transported from the cell mainly through MRP1 and MRP4. The same types of transporters (ABC and SLC transporters) are present on the BBB (Grube et al., 2018). It is assumed that there is a predominant influx of steroid sulfates from the circulation across the BBB to the brain due to huge concentration gradient (Wang et al., 1997; Qaiser et al., 2017; Grube et al., 2018).

The most important conjugated NAS include DHEAS, PregS and conjugated 5α/β reduced pregnane and androstane isomers. The steroid conjugates in the blood dominate over their free counterparts from one to four orders of magnitude. On the other hand, the levels of unconjugated DHEA, Preg, and reduced 5α-pregnane steroids were found in substantially higher amounts in all brain regions compared to their conjugated counterparts (Weill-Engerer et al., 2002). Looking more closely to individual brain regions, the highest levels of DHEAS were found in striatum, hypothalamus and cerebellum and those of PregS in striatum and hypothalamus (Weill-Engerer et al., 2002).

DHEAS exert neuroprotective, neuroexcitatory, antidepressant and memory enhancing effects. Together with DHEA, DHEAS has anti-inflammatory and immunomodulating effects, positive effects on neurite growth, neurogenesis and neuronal survival as described earlier (reviewed in (Maninger et al., 2009; Stárka et al., 2015)).

PregS, similarly as DHEAS, is an excitatory NAS. It has enhancing effects on the adult hippocampal neurogenesis, neurite growth and the survival of newborn neurons (Xu et al., 2012). It plays a role in the modulation of memory and learning (Smith et al., 2014; Wong et al., 2015).

## Mechanism of Action of Neuroactive Steroids

### Genomic Action

Steroids can surpass the BBB from periphery to the brain either by passive diffusion (unconjugated steroids) or in cooperation with transporter proteins mentioned above (steroid conjugates). Unconjugated or deconjugated steroids can bind to intracellular receptors in the brain and act as transcriptional factors regulating gene expression (Rupprecht et al., 1996). This action may be preceded by intracellular metabolization of the steroids (Rupprecht, 2003). This genomic effect is generally delayed in the onset, because it is limited by the rate of protein synthesis, but it has longer lasting effects.

### Non-Genomic Action

In addition to this classical genomic effect, NAS (unconjugated as well as conjugated) are able to bind to various membrane receptors where they can act as their allosteric modulators and induce fast nongenomic effects in values from milliseconds to seconds (McEwen, 1991; Joëls, 1997). Both fast and delayed actions can potentially/subsequently alter membrane excitability (Joëls, 1997).

The mechanism of action of NAS lies mainly in affecting the excitability of the nervous cells. They are able to modulate permeability of ion channels. In the CNS, the best-known receptors modulated by NAS are type A γ-aminobutyric acid (GABA_A_) receptors and glutamate receptors including NMDA receptors, α-amino-3-hydroxy-5-methyl-4-isoxazolepropionic acid (AMPA) receptors and kainate receptors (Joëls, 1997; Wu and Chen, 1997; Wu et al., 1998; Yaghoubi et al., 1998; Beyenburg et al., 2001; Rupprecht, 2003; Shirakawa et al., 2005). Furthermore, interactions of NAS with glycine, transient receptor potential (TRP), nicotinic acetylcholine, muscarinic acetylcholine, sigma (σ)-receptors and several types of voltage-gated calcium channel were reported (Klangkalya and Chan, 1988; Valera et al., 1992; Monnet et al., 1995; Hu et al., 2007; Xu et al., 2008; Majeed et al., 2012; Bukanova et al., 2020).

Stereoselectivity is an important property when binding to GABA_A_ and NMDA receptors. The presence of a 3α-hydroxy group on the A ring is necessary for the positive modulation of GABA_A_ receptor. The GABA_A_ receptor positive modulators include 3α-pregnane steroids (Majewska et al., 1986), including the tetrahydrodeoxycorticosterone (THDOC) isomers, as well as 3α-androstane metabolites (Turner et al., 1989; Kaminski et al., 2005). These substances act via increasing the frequency and opening time of the GABA_A_ receptor (for chloride ions). The influx of chlorides into nerve cells reduce the neuronal excitability. Thus, these substances are neuroinhibitory and exhibit sedative, hypnotic, anesthetic, anxiolytic and anticonvulsant properties. The 3β-pregnane steroids (Wang et al., 2000), and particularly their conjugates (Park-Chung et al., 1999) as well as the Δ^5^ steroid sulfates (PregS, DHEAS) act as negative GABA_A_ receptor modulators and activate the neuronal activity in this way. Nanomolar concentrations of steroids are necessary for the positive modulation of GABA_A_R, while the antagonists act only in micromolar amounts (Park-Chung et al., 1999). Steroid modulators of selected membrane receptors are shown in Table 1.

Positive and negative steroid modulators are also known for the NMDA receptor. Positive modulators, upon binding to the receptor, increase the influx of calcium ions into the cell and thus cause activation of the neuron. These include Δ^5^ steroid sulfates (Wu et al., 1991; Irwin et al., 1992) and polar conjugates of 5α-pregnane steroids (predominantly sulfates) (Weaver et al., 2000). Polar conjugates of 5β-pregnane steroids have the opposite effect (Park-Chung et al., 1994; Yaghoubi et al., 1998; Weaver et al., 2000). These substances are summarized in Table 1 together with positive and negative modulators of further receptors.

## Conjugation and Deconjugation

**FIGURE 2:**
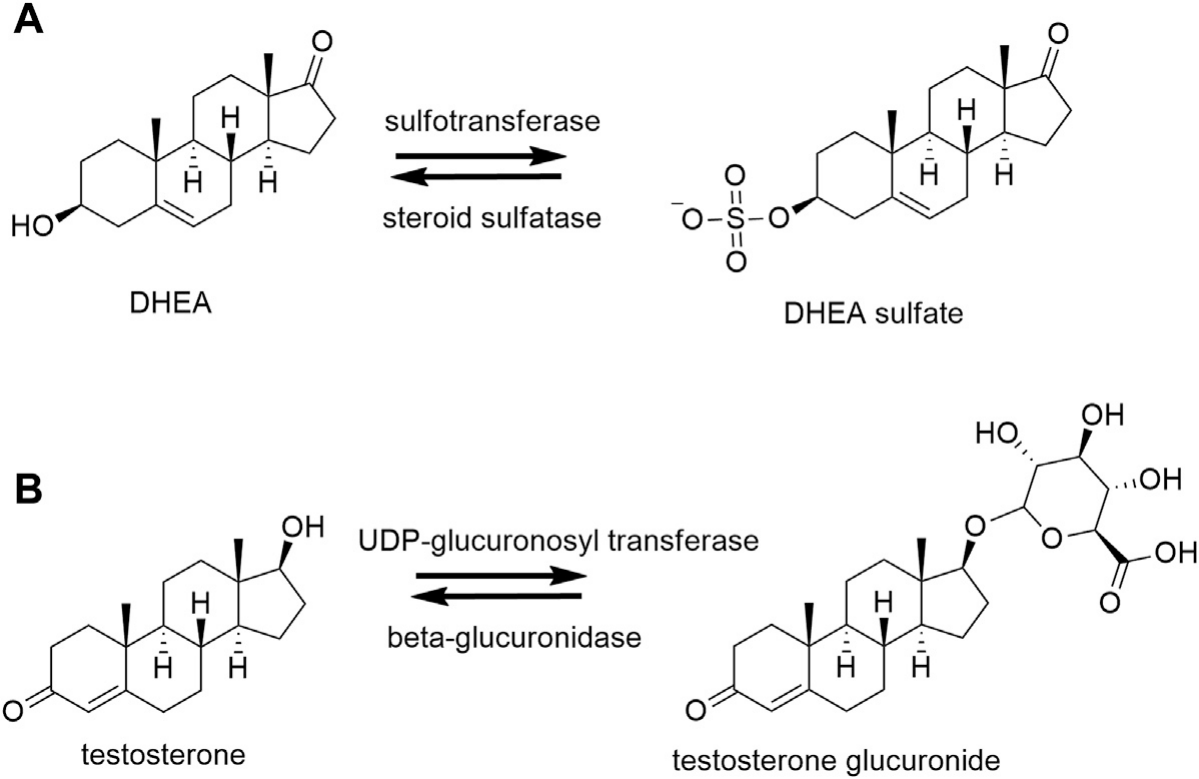
Conjugation and deconjugation reactions. **(A)** Example of sulfation and desulfation reactions of dehydroepiandrosterone (DHEA) by sulfotransferase and steroid sulfatase, respectively. **(B)** Example of glucuronidation and deglucuronidation of testosterone by uridine 5′-diphospho (UDP)-glucuronosyltransferase and β-glucuronidase, respectively.

Steroids can be conjugated either by sulfotransferases (SULTs) to form sulfates or by uridine 5′-diphospo (UDP)-glucuronosyltransferases to form glucuronides (Figure 2). These processes increase their polarity and water solubility and facilitate the excretion in urine and bile (Schiffer et al., 2019). Furthermore, sulfates having greater half-lives than their unconjugated counterparts, also function as a steroid pool in the circulation (mainly DHEAS and estrone sulfate). Finally, sulfated steroids (e.g., PregS and DHEAS) may modulate some ligand-gated ion channels in the CNS as was described above.

The form of conjugation (sulfation/glucuronidation) depends on the structure of the steroid. Sulfation occurs mainly in ∆^5^ steroids such as DHEA, pregnenolone and estrogens (estrone). Alternatively, glucuronidation takes place mainly in the phase 2 metabolism of ∆^4^ steroids (Mueller et al., 2015; Schiffer et al., 2019).

### Sulfotransferases

Sulfation takes place by two-step enzymatic reactions; 1) activation of the sulfate group in the form of 3′phosphoadenosine-5′-phosphosulfate (PAPS) by PAPS synthase and 2) transfer of activated sulfate on hydroxyl group of the steroid by SULT (Schiffer et al., 2019). Five cytoplasmic SULTs are known to be involved in steroid metabolism–SULT1A1, SULT1E1, SULT2A1 and 2 isoforms of SULT2B1 (SULT2B1a a SULT2B1b) (Hempel et al., 2000; Fuda et al., 2002; Chang et al., 2004; Gamage et al., 2005). They possess broad substrate specificity; instead of steroid sulfation they can also metabolize phenolic drugs and catecholamines (SULT1A), thyroid hormones (SULT1B) and sterols (SULT2B) (Strott, 2002). Regarding steroids, they can have preferred substrate e.g. SULT1E1 preferentially sulfates estrogens and SULT2A1 most androgens and pregnenolone (reviewed in (Mueller et al., 2015)) and SULT2B1 isoforms stereo-specifically sulfate 3β-hydroxysteroids (e.g., pregnenolone, cholesterol) (Meloche and Falany, 2001; Fuda et al., 2002). SULTs are ubiquitous enzymes with the highest concentrations found in the liver and intestine compared to the kidney and lung (Riches et al., 2009). SULT2A1 is strongly expressed in adrenal *zona reticularis*, *zona fasciculata* and the liver. SULT2A1 probably has a dual function: in adrenals it is responsible for massive sulfation of DHEA and pregnenolone and it detoxifies xenobiotics in the liver (Falany and Rohn-Glowacki, 2013).

Regarding the detailed expression of SULTs in brain, SULT1A1 expression was detected in several brain regions (Salman et al., 2009). SULT2A1 was detected exclusively in the thalamus and hypothalamus (Shimizu and Tamura, 2002), it was not detected in other brain regions. These findings are in agreement with the those of Salman et al. who analyzed specimens of prefrontal cortex, hippocampus, and cerebellum (Salman et al., 2011). The results on SULT2B1b expression are ambiguous. No mRNA expression of SULT2B1b in the brain was reported by some authors (Her et al., 1998; Meloche and Falany, 2001), conversely, SULT2B1b mRNA expression was reported in a large number of sections of the human brain by other research groups (Shimizu and Tamura, 2002; Salman et al., 2011). Although SULT2B1a mRNA was detected by Salman et al. (but not by (Shimizu and Tamura, 2002)), no SULT2B1a immunoreactive protein was observed. SULT1E1 expression was not found in human brain in one study (Salman et al., 2011). Taken together, some types of SULTs are apparently available in the brain and therefore may play a crucial role in neurosteroid sulfation and control.

### Steroid Sulfatase

Sulfation is reversible; steroid sulfate can be desulfated by steroid sulfatase (EC 3.1.6.2, STS, aryl sulfatase C) to unconjugated steroids in the target tissue. STS belongs to the sulfatase family containing 17 members, while only STS uses steroids as substrates (Diez-Roux and Ballabio, 2005). It is expressed as a membrane associated enzyme. Immunohistochemical techniques showed localization mainly in the rough endoplasmic reticulum. Furthermore, it is localized in Golgi cisternal, trans-Golgi reticulum, plasma membranes, endosomes and lysosomes (Willemsen et al., 1988; Stein et al., 1989). The main substrates for STS are estrone sulfate, DHEAS, PregS and cholesterol sulfate (Mueller et al., 2015). It is most abundantly expressed in placenta, however, it is believed to be ubiquitous in low amounts (Reed et al., 2005) in other tissues including the brain (Iwamori et al., 1976; Steckelbroeck et al., 2004). Within the brain the high activity of STS was observed in the thalamus, hypothalamus, hippocampus and temporal lobe (Perumal and Robins, 1973; Steckelbroeck et al., 2004). Furthermore, reports from rat studies indicate that STS is present in brain capillaries of BBB and can rapidly desulfate DHEAS that comes across the BBB from the circulation (Nicolas and Fry, 2007; Qaiser et al., 2017).

Mutations in the gene for STS result in X-linked ichthyosis, which is a rare skin disorder. Several extracutaneous manifestations have been associated with this disease including corneal opacities, cryptorchidism and chondrodysplasia punctata (Fernandes et al., 2010). While STS is expressed in various brain structures, it can be hypothesized that alterations in brain function might be present. Neurological (mental retardation, epilepsy), neurodevelopmental (attention deficit hyperactivity disorder, autism) and mood disorders are more frequent in individuals with X-linked ichthyosis compared to the general population (Fernandes et al., 2010; Chatterjee et al., 2016). From neuroanatomical point of view, structural changes in basal ganglia (reduced right putamen and pallidum volume, and left accumbens volume) in female carriers were reported (Brcic et al., 2020). The described structural changes seem to be also involved in the degeneration in PD. The recent results of Hickman et al. (2022) showed that neurodevelopmental and neurodegenerative diseases may overlap. The genetic alterations which can lead to AD and PD were also described in detail by the authors (Hickman et al., 2022).

### Uridine 5′-Diphospho-Glucuronosyl Transferases and β-glucuronidase

For completeness, except for sulfation, steroids may be conjugated to steroid glucuronides by UDP-glucuronosyl transferases (UGT), with the UGT1 a 2. However, this process is irreversible in the humans except for the activity of certain gut bacteria that possess β-glucuronidase activity (Schiffer et al., 2019). The process of glucuronidation is used for excretion through bile and urine and steroid glucuronides are not neuroactive.

### Balances Between Conjugated and Unconjugated Neuroactive Steroids

Balances between conjugated and unconjugated NAS, except for adrenal sulfated Δ^5^ steroids, are ensured mainly by hepatic STS, SULTs, and possibly UDP-glucuronosyltransferases. Sulfation pathways prevail in healthy brain, colon, adrenal, and kidney while desulfation dominates in breast, ovary, prostate, testis, placenta and uterus (Mueller et al., 2015). These balances may be of great importance as unconjugated and sulfated steroids act in many cases in opposite ways on the same receptors.

For example, 5α/β-reduced metabolites with a hydroxy group in the 3α-position are positive modulators of GABA_A_ receptors. However their sulfation reverses their action from the positive to negative modulation (Park-Chung et al., 1999). Additionally, the sulfation in the 3α-position enables steroid modulation at NMDA receptor. These data suggest that sulfation and desulfation might be the critical point in steroid regulation of GABAergic and glutamatergic neurotransmission (Park-Chung et al., 1994; Park-Chung et al., 1999). Sulfation also increases the polarity of substances and contributes to their better solubility in the circulation, while rather inhibiting their passage through the BBB.

## Sulfation in Neurodegenerative Diseases

Neurodegenerative diseases including multiple sclerosis (MS), Alzheimer’s disease (AD) and Parkinson’s disease (PD) are generally characterized by progressive alterations in the brain and the spinal cord (Bianchi et al., 2020). These diseases are usually accompanied by neuroinflammation, which may contribute to neurodegeneration (Yilmaz et al., 2019). In addition, neurosteroid synthesis can be affected by neuroinflammation and *vice versa*, neuroactive steroids can influence neuroinflammation. Alterations in neurosteroids (mainly unconjugated) in the aforementioned neurogenerative diseases were thoroughly reviewed in 2011 by Luchetti et al. (2011). The current review presents the results of some of the later published studies.

### Alzheimer’s Disease

The pathophysiology of Alzheimer’s disease (AD) is characterized by a formation of extracellular amyloid plaques in the cortex and limbic system, aggregation of hyperphosphorylated τ-protein causing intracellular neurofibrillary tangles and is often accompanied by reactive microgliosis and loss of synapses, cholinergic, serotonergic, and noradrenergic function together with glutamatergic dysfunction (López and DeKosky, 2008; Reitz and Mayeux, 2014; Vaňková et al., 2016). The clinical picture is formed by memory loss and cognitive impairment that are often accompanied by various neurological and psychiatric symptoms (López and DeKosky, 2008).

The study of Vaňková et al. (2016) examined 16 AD female patients and 22 sex- and age-matched heathy controls. The measurement of 30 unconjugated steroids and 17 conjugates in the circulation of the AD patients showed altered various steps of the steroidogenesis. The authors found a shift from conjugated to free (unconjugated) steroids in the AD patients probably due to the reduced SULT2A1 activity in the liver and the adrenal *zona reticularis*. Despite this finding, the relative overproduction of C21 steroids was sufficient to maintain higher levels of sulfates such as PregS, allopregnanolone sulfate, conjugated pregnanolone and conjugated 5β-pregnane-3α,20α-diol in AD patients (Vaňková et al., 2016). The same findings of attenuated sulfotransferase activity measured as the ratio between conjugated and corresponding unconjugated steroids were confirmed in the cohort of 18 male and 16 female AD patients compared to corresponding age- and gender-controls (Vaňková et al., 2015).

Generally, lower plasma levels of DHEAS in AD patients are reported when compared with control subjects (Näsman et al., 1991; Genedani et al., 2004; Aldred and Mecocci, 2010; Pan et al., 2019). Besides the lower plasma levels of DHEAS, Yanase *et al.* also found lower values of DHEAS/DHEA ratio in patients with AD and cerebrovascular dementia. This indicates decreased peripheral sulfotransferase activity in dementias in general (Yanase et al., 1996). A recent meta-analysis showed lower DHEAS plasma levels in AD patients (Pan et al., 2019), in accordance with reduced sulfotransferase activity in AD patients. Furthermore, AD patients with higher DHEAS plasma levels were more successful in some memory tasks than patients with lower DHEAS levels (Carlson et al., 1999). Moreover, the results from a prospective study supported the role of lower DHEAS as a risk factor for AD (Hillen et al., 2000) and indicates attenuated sulfotransferase activity even before the development of the disease.

The conclusions drawn from the examinations of the circulating steroids are in line with the observations in CSF, where the steroid levels may also reflect the steroid production and metabolism in the brain. Higher DHEA but lower DHEAS levels in CSF were reported in patients with AD and vascular dementia (Kim et al., 2003). This indicates the attenuated sulfation in the brain of patients with dementia and suggests that DHEA itself does not protect from neurodegeneration. However, it may be a compensatory mechanism against the neurodegenerative process. Lower DHEAS and PregS levels were found in certain brain regions of AD patients examined *postmortem* together with negative correlation of these sulfates with key proteins involved in plaque formation (Weill-Engerer et al., 2002). These results allow to speculate about the neuroprotective role of sulfated steroids and/or the sulfation in AD.

Data from genome wide association study (GWAS) showed downregulation of *STS* gene in patients with AD (Wu et al., 2019). Further GWAS revealed eight independent single nucleotide polymorphisms (SNPs) associated with serum DHEAS concentration. The results elucidated a certain role for *SULT2A1* gene which provides information about key mechanisms of degeneration and aging (Zhai et al., 2011).

STS inhibitors influence the ratio between sulfated and non-sulfated steroids which subsequently modulate brain function. Administration of the STS inhibitor DU-14 to rats increased plasma DHEAS, decreased plasma DHEA and enhanced hippocampal acetylcholine release and memory (Rhodes et al., 1997). Additionally, significantly higher levels of serotonin in the striatum and hippocampus were reported in mice lacking *STS* gene (Trent et al., 2012). Therefore, steroid sulfation may influence processes in the hippocampus (one of the earliest sites affected in AD (Braak et al., 1993; Mu and Gage, 2011)) by multiple mechanisms, including an alteration of the cholinergic and serotoninergic signaling (Trent et al., 2012).

A recent study of Pérez-Jiménez *et al.* showed that the treatment with STS inhibitor STX64 (Irosustat) resulted in higher pool of sulfated steroids and subsequently increased longevity, improved cognitive symptoms and plaque formation in a chronic AD murine model (Pérez-Jiménez et al., 2021). Furthermore, the use of another STS inhibitor DU-14 in chronic AD murine model decreased the cognitive deficits in spatial learning and memory and protected hippocampal synaptic plasticity (Yue et al., 2016). These data may be of importance for treatment of age-related diseases such as AD in humans. Interestingly, the concept of inhibition of STS has been longer studied in the context of hormone dependent cancers (reviewed in (Foster, 2021)). The use of STS inhibitor STX64 in phase II clinical trial was efficient for the treatment of breast cancer with an acceptable safety profile (Palmieri et al., 2017).

### Parkinson’s Disease

Parkinson’s disease (PD) is a neurodegenerative disorder that predominantly presents in later life with bradykinesia and at least one other symptom of resting tremor or rigidity. People with PD can develop cognitive impairment, including memory loss and dementia. Parkinson’s dementia is the second most common dementia after AD and is characterized by neurodegeneration in areas related to motor control, coordination and cognitive function (Mendell and MacLusky, 2018). These features are caused by a massive loss of dopaminergic neurons in the *substantia nigra pars compacta* and consequent striatal dopamine deficiency (di Michele et al., 2013). The pathological hallmark is α-synuclein aggregation into intraneuronal inclusions named Lewy bodies (Mahul-Mellier et al., 2020). Prevalence of PD is twice higher in men than in women, however, women have higher mortality rate and faster progression of the disease (Cerri et al., 2019).

The mechanism how steroid sulfation may be involved in the pathophysiology of PD might lie in the modulation of dopaminergic neurons in substantia nigra that are also under control of the excitatory glutamatergic and inhibitory GABAergic systems (Cobb and Abercrombie, 2002).

The few studies which examined alterations in neurosteroid levels in PD were mostly focusing on unconjugated NAS such as allopregnanolone (di Michele et al., 2003). While DHEAS was found to be lower in AD and vascular dementia (Yanase et al., 1996), no changes were observed in DHEAS levels in PD patients when compared with healthy controls in circulation (Genedani et al., 2004). However, the expression of SULT2B1 was downregulated in *substantia nigra* of PD patients with no changes in sulfatase expression (Luchetti et al., 2010). These results indicate that there can be brain region-specific changes in the bioavailability of neuroactive sulfate steroids, which may consequently affect the balance between GABAergic and glutamatergic systems and finally worsen the degeneration of dopaminergic cells (di Michele et al., 2013). The question is whether the decreased levels of neuroprotective NAS contribute to the neurodegeneration or they may be the primary cause of it (Luchetti et al., 2011).

STS inhibitors as well as mutations in *STS* gene were tested in *Caenorhabditis elegans* PD model. Mutation in STS gene or administration of STX64 improved mobility and decreased the number of α-synuclein aggregates (Pérez-Jiménez et al., 2021) indicating that the use of STS inhibitors in PD could be also a promising treatment option.

### Multiple Sclerosis

Multiple sclerosis (MS) is an autoimmune inflammatory and demyelinating disease of the central nervous system with symptoms occurring most often between age of 20–40. Clinical representation is variable with either cognitive and/or motor impairment depending on the localization of the lesion(s). Compared to PD, the prevalence of MS is, on the contrary, at least twice higher in women than in men (Luchetti et al., 2014).

Several studies examined changes in unconjugated NAS in the brain of MS patients. DHEA and allopregnanolone levels in the white matter of MS patients were reported to be decreased (Noorbakhsh et al., 2011; Boghozian et al., 2017). Gender-dependent changes in progesterone and estradiol synthesis and signaling in MS lesions have been described where estrogen pathways were predominantly activated in MS lesions of male patients whereas progesterone pathways were predominantly activated in MS lesions of female patients (Luchetti et al., 2014). In CSF of MS patients, increased levels of pregnenolone and DHEA compared to control groups were reported (Orefice et al., 2016). Additionally, an increase in pregnenolone and isopregnanolone levels together with a decrease in dihydroprogesterone and allopregnanolone levels in CSF of male MS patients were observed in the study of Caruso *el al*. (Caruso et al., 2014). Dysregulation in biosynthesis of allopregnanolone of MS patients and its potentially therapeutic role was recently reviewed (Melcangi and Panzica, 2014; Noorbakhsh et al., 2014; Balan et al., 2019). To the best of the authors’ knowledge, no information about sulfated steroids and sulfation pathways in MS patients in brain tissue or CSF is available.

At the peripheral level, low plasma testosterone was found in men as well as in women with MS (Foster et al., 2003; Tomassini et al., 2005; Foroughipour et al., 2012). A recent study of Cheng *et al.* found changes in isopregnanolone and allopregnanolone plasma levels when comparing patients with relapsing-remitting MS and patients with a clinically isolated syndrome (Cheng et al., 2021).

In a study of Kancheva *et al.*, 51 steroids and steroid polar conjugates were analyzed in 12 women with MS and 6 sex-and age-matched controls in follicular phase of menstrual cycle. Intriguingly, the results showed higher levels of C21 steroids including pregnenolone and 3α/β pregnane isomers and also higher levels of conjugated steroids such as PregS, 20α-dihydropregnenolone sulfate, conjugates of 3α/β pregnane isomers and certain bioactive C19 steroids (androsterone, 5-androsten-3β,7α,17β-triol) in MS patients (Kanceva et al., 2015) similarly as in AD patients (Vaňková et al., 2016). The altered levels of these steroids may influence neural activity by interacting with various neurotransmitter receptors on a neuronal membrane and affect the balance between neuroprotection and excitotoxicity.

The results regarding DHEAS levels in MS are ambiguous. Levels of DHEAS did not differ between MS and control patients in the above mentioned study of Kanceva et al. (2015). On the other hand, two studies found lower levels of DHEAS in MS in comparison with healthy subjects (Ramsaransing et al., 2005; Foroughipour et al., 2012) and one study found higher DHEAS in MS patients (Ysrraelit et al., 2008). Further, lower serum levels of DHEAS and DHEA were found in patients with fatigue in comparison with those without fatigue within the progressive form of MS (Téllez et al., 2006).

The research also reveals the role of glutamate in the pathophysiology of MS. Higher glutamate concentrations were found in acute lesions and normal-appearing white matter (Srinivasan et al., 2005) and appear to contribute to the progression of the disease (Azevedo et al., 2014). GWAS which used *in vivo* glutamate concentration as a quantitative trait discovered that several SNPs are associated with glutamate concentrations. One of these SNPs is rs794185 (*p* < 6.44 × 10^-^7), which is within the *sulfatase modifiying factor 1 (SUMF1)* gene (Baranzini et al., 2010). SUMF1 is an essential factor for sulfatase activities, including the one of STS. The dysregulation of SUMF1 can lead to its diminished activity (Fraldi et al., 2007). Subsequently, an imbalance between conjugated and unconjugated steroids that can modulate glutamatergic receptors occurs.

The available data concerning the NAS and particularly sulfation pathways in association to MS in humans are limited. Therefore, the data from animal models may be helpful. In the mouse model of MS–experimental autoimmune encephalomyelitis (EAE)—a protective effect of unconjugated DHEA on the development and severity of the EAE was repeatedly reported (Du et al., 2001; Aggelakopoulou et al., 2016). Similarly, DHEAS administration to mice ameliorated EAE severity and improved neurological outcomes in EAE, possibly through anti-inflammatory effects (Boghozian et al., 2017).

## Conclusion

The expression of SULTs and STS is brain region specific, the explanation of these differences across brain region remains to be elucidated. The balance between steroid sulfation and desulfation is critical to maintaining the balance between unconjugated and conjugated steroids, especially when their CNS action is reversed. In neurodegenerative diseases such as Parkinson’s disease and Alzheimer’s disease the reduced SULT expression and lower levels of steroid sulfates were reported in the brain. In multiple sclerosis, no information about steroid sulfation and steroid sulfate levels is available yet. However, as with other neurodegenerative diseases, changes in steroid sulfation could be expected. Changes in sulfation in neurodegenerative diseases occur also at the peripheral level as mainly documented by changed ratios of conjugated steroids to their unconjugated counterparts. These alterations may subsequently affect the neuronal activity in the CNS, as the circulating unconjugated steroids and to a lesser extent also the steroid conjugates from periphery surpass the BBB and enter the brain. Therefore, further research of the steroid sulfation in periphery and in brain deserves attention.

Future studies aiming to decipher the relative contributions of the effects of steroid sulfation on neurodegeneration by neurochemical/inflammatory/developmental/general health process may choose different approaches to answer these scientific questions. Animal experiments may be one of the options. However, the limitations of the animal studies are due to the different steroidogenesis in humans and commonly used laboratory animals. In fact, compared to primates, laboratory rodents have negligible steroid sulfate production and generally very different adrenal steroidogenesis (Schuler, 2021). Another route may be GWAS or transcriptomic studies, which are promising approaches suitable for studying the genome or transcriptome and the pathophysiology of human diseases. However, their disadvantage might be the need for a larger sample size (Hong and Park, 2012). Finally, steroidomic studies may also be designed to explore the mutual association between the metabolites studied.

STS inhibitors are gaining increased attention in the context of aging and age-related diseases. Use of STS inhibitors in animal studies shows promising results in increasing longevity and reducing protein aggregation in protein aggregation diseases such as AD and PD (Pérez-Jiménez et al., 2021). Based on the promising animal results clinical studies in humans are justifiable and warranted.
